# Cost-Utility Analysis of Berberine Chemoprevention for Colorectal Cancer After Polypectomy

**DOI:** 10.7759/cureus.61030

**Published:** 2024-05-24

**Authors:** Shuai Yuan, Tian Zhang, Yingyu Wu, Yun Lu, Feng Chang, Yumei Zhu

**Affiliations:** 1 School of International Pharmaceutical Business, China Pharmaceutical University, Nanjing, CHN

**Keywords:** cost-effectiveness, polypectomy, colorectal adenomas, colorectal cancer, chemoprevention, berberine

## Abstract

Background

Chemoprevention, such as berberine, has been developed as an alternative or complementary strategy to colonoscopy surveillance and has shown promise in reducing the morbidity and mortality of colorectal cancer. This study aims to evaluate the cost-effectiveness of berberine for postpolypectomy patients from the US third-party payer.

Methods

A Markov microsimulation model was developed to compare the cost and efficacy of berberine to no intervention, colonoscopy, and the combination of berberine and colonoscopy in postpolypectomy patients.

Results

After simulating 1 million patients, the study found that colonoscopy alone had a mean cost of $16,391 and mean quality-adjusted life-years (QALYs) of 16.03 per patient, whereas adding berberine slightly reduced the mean cost to $15,609 with a mean QALY of 16.05, making it a dominant strategy. Berberine therapy alone was less effective than colonoscopy alone, with a higher mean cost of $37,480 and a mean QALY of 15.32 per patient. However, berberine therapy was found to be a dominant strategy over no intervention.

Conclusions

Adding berberine to colonoscopy is the most cost-saving and effective approach for postpolypectomy patients. For patients who refuse or have limited access to colonoscopy, berberine alone is likely to be a dominant strategy compared to no intervention.

## Introduction

Colorectal cancer (CRC) ranks third among all cancer types, with approximately 150,000 new cases diagnosed annually in the United States (US). Up to 95% of CRC cases arise from precancerous adenomatous polyps through a series of well-defined genetic and histopathological changes, referring to the adenoma-carcinoma sequence [1, 2]. Therefore, the early detection and removal of precancerous lesions by colonoscopy, the most commonly performed method for primary screening, significantly decreases the incidence and mortality of CRC. However, colorectal polyps often recur at a rate of 20% to 50% in patients who have undertaken polypectomy, especially those with advanced histology [3,4]. Intensive monitoring, like regular postpolypectomy surveillance by colonoscopy, has been recommended for such patients and has become a common feature of CRC prevention. However, there are barriers of poor adherence, invasive harms, and high cost for surveillance colonoscopy [5,6], leading to the development of chemoprevention, such as aspirin, cyclooxygenase-2 inhibitors, calcium, and many other agents, as alternative or complementary strategies to surveillance colonoscopy [7-11]. Among these chemopreventive agents, few are recommended as evidence-based prevention or cost-effective alternatives due to inconclusive results, short observation time, and side effects [12-15], sustaining the interest in searching for other viable chemopreventive options with cost-effectiveness.

Recent evidence from a double-blind, randomized, placebo-controlled trial (NCT02226185) suggested berberine supplementation reduced the recurrence risk of colorectal adenomas (CRA) (unadjusted relative risk ratio 0.77, 95% confidence interval (CI) 0.66-0.91) after polypectomy without serious adverse events [16]. The compound, whose chemical name is benzyltetrahydroxyquinoline, is an isoquinoline alkaloid now synthesized but was initially extracted from several plants such as *Berberis *(Oregon grape), *Hydrastis canadensis* (goldenseal), and *Berberis vulgaris* (barberry). As a common ingredient in Ayurvedic and Chinese medicines, berberine has been used for centuries for its perceived antimicrobial and antidiarrheal properties. More recently, reports have emerged trumpeting its potency to inhibit CRC in carcinogen-induced cancer models [17], genetically engineered mouse models [18], as well as syngeneic and xenogeneic transplant models [19, 20]. This intriguing anti-cancer effect has been associated with multiple routes in the development of CRC, including increased apoptosis, reduced inflammation, as well as attenuated oxidative stress and microRNA levels [21].

Since berberine is cheap, accessible, and exhibits a good risk-benefit profile relative to other chemotherapies, we postulated that berberine chemoprevention might represent a cost-effective alternative for individuals who have undergone polypectomy. The recent randomized trial provides a unique opportunity to weigh the value of berberine chemoprevention in an adjuvant or secondary CRC prevention setting [16]. Thus, the present study examined the cost-effectiveness of berberine chemoprevention with or without surveillance colonoscopy in postpolypectomy patients from the perspective of the US third-party payer. Moreover, the study explored the cost-effectiveness of berberine chemoprevention combined with surveillance colonoscopies of routine or adjusted intensity.

## Materials and methods

General study assumptions

The present study developed a Markov microsimulation model in TreeAge Pro statistical software version 2019 R1 (TreeAge Software, Williamstown, Massachusetts) to estimate the health and cost outcomes of CRC prevention strategies in postpolypectomy patients over 50 years old. A total of four interventions were evaluated in the model: 1) no intervention (no berberine or colonoscopy surveillance); 2) berberine chemoprevention without further colonoscopy surveillance; 3) routine colonoscopy surveillance based on the grade of polys; and 4) a combination of berberine chemoprevention and routine colonoscopy surveillance. This study compared each of the latter three interventions to the option of no intervention. Patients entered the model and received interventions from 50 to 75 years of age, followed until 100 or death. In detail, the daily administration of berberine chemoprevention was 600 mg, according to the clinical trial. The model simulated routine colonoscopy surveillance on a three-year cycle since the initial colonoscopy in the third year in case of high-grade polyps (≥10 mm in size, with features of high-grade dysplasia or villous histology). The cycle would be extended to five intervals if a low-grade polyp (<10 mm in size, without the features of high-grade dysplasia or villous histology) or no polys were detected.

**Figure 1:**
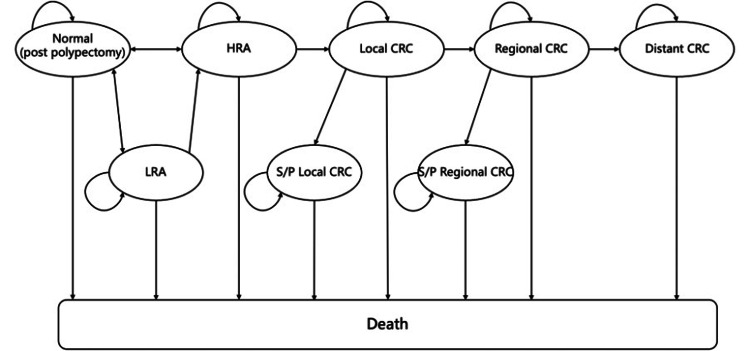
Markov model structure HRA - high-risk adenomas; LRA - low-risk adenomas; CRC - colorectal cancer; S/P - status post

The model simulated seven health states to present the natural history of sporadic colorectal neoplasm, including 1) no polys/normal colonic mucosa, 2) low-risk adenoma, 3) high-risk adenoma, 4) local CRC, 5) regional CRC, 6) distant CRC, and 7) death (Figure 1). To minimize the effect of statistical fluctuations on the outcomes, the model simulated 1,000,000 patients after polypectomy. All patients entered the model in the no polys/normal colonic mucosa state and could transition to another. The transition between health status occurred annually until death. The study superimposed berberine and colonoscopy on the natural history module, resulting in the prevention of CRA recurrence and early detection of CRA and CRC, respectively. Once CRA recurrence and CRC are diagnosed with colonoscopy, they can be treated, resulting in postpolypectomy or stage-specific survival.

Transition probabilities

The annual transitions among health states, shown in Table 1, were derived from clinical trials and previous literature. The model considered different compliance rates with surveillance colonoscopy and berberine following polypectomy. In the base case, the compliance was 60% for those postpolypectomy patients who use colonoscopy according to previous trials [22, 23]. Berberine compliance was 80%, which was in line with previous studies on preventing postpolypectomy adenoma recurrence with chemoprevention agents such as aspirin and calcium [24, 25]. Recurrence rates of low-risk and high-risk adenomas were obtained from the double-blind, randomized, placebo-controlled trial (NCT02226185). Based on this trial, low-risk adenomas had appeared after two years among 33% and 41% of patients for the berberine group and the placebo group, respectively, while the proportion would drop to 3% and 6% in the case of high-risk adenomas 16. The model assumed the constant incidence rates in the two-year probabilities, applying the following methods to calculate the annual recurrence rate of low- or high-risk adenomas: 1) rate = -ln (1-p)/t and 2) probability=1- exp (-rt), where p is the probability, t is the time taken to develop the probability, and r is the rate. Transition probability from low- to high-risk adenoma and from high-risk adenoma to CRC in the berberine group was not available in the clinical trial. Therefore, the study assigned a realistic estimate of the placebo group to the berberine group in the base case according to previous literature [26,27]. The effectiveness of colonoscopy depends on the efficacy of colonoscopy plus polypectomy to prevent CRCs [26]. Since the berberine treatment induced a few severe complications (constipation, 1%; hepatic dysfunction, less than 0.5%), the model only simulated the complications for colonoscopy [28]. CRC incidence and specific deaths were extracted from the literature [29]. The CRC-specific mortality reflected the mortality rates with varied CRC stages. Besides, mortality of the complication and other-cause mortality were applied. The model obtained the mortality of the complication also from previous literature. Of those who had complications during colonoscopy, there was a 5.8% and 6.0% mortality rate of perforation and major bleeding events, respectively [30, 31]. The other-cause mortality was modeled as age-dependently based on the US life tables [32].

Cost and utility inputs

The study included direct medical costs from the perspective of a third-party payer in the US. All costs were adjusted to 2022 US dollars using the medical component of the consumer price index and discounted at a rate of 3% per year [33]. The study used payment rates derived from Medicare reimbursement rates and CRC care costs for persons aged 65 years and over and commercial payment rates for persons aged between 50 and 64, which reflected different costs in commercial insurance and medical payments. As for patients aged <65 years, the CRC care and related complication costs were adjusted by 1.35-fold of the mean ratio of commercial to Medicare payment rates for colorectal costs with patients aged ≥65 years [34]. Health state utilities for CRC by stage were obtained from previous literature [35,36], as listed in Table 1.

**Table 1:** Model parameters HRA - high-risk adenomas; LRA - low-risk adenomas; CRC - colorectal cancer; S/P - status post; PSA - probabilistic sensitivity analysis

Parameters	Baseline value	Range	Distribution	Source
Annual transition probabilities, %				
S/P polypectomy to LRA in berberine group	18.15	16.34 -19.97	Beta	Chen et al. [16]
S/P polypectomy to HRA in berberine group	1.51	1.36- 1.66	Beta	Chen et al. [16]
LRA to HRA in berberine group	8.90	8.34 - 9.45	Beta	Veettil et al. [26]
HRA to local CRC in berberine group	5.00	4.50 - 5.50	Beta	Ladabaum et al. [27]
S/P polypectomy to LRA in placebo group	23.19	20.87 - 25.51	Beta	Chen et al. [16]
S/P polypectomy to HRA in placebo group	3.05	2.74 - 3.35	Beta	Chen et al. [16]
LRA to HRA in placebo group	8.90	8.34 - 9.45	Beta	Veettil et al. [26]
HRA to local CRC in placebo group	5.00	4.50 - 5.50	Beta	Ladabaum et al. [27]
Local CRC to regional CRC	56.00	50.40 - 61.60	Beta	Veettil et al. [26]
Regional CRC to distant CRC	63.00	56.70 - 69.30	Beta	Veettil et al. [26]
Symptomatic presentation of localized CRC	22.00	19.80 - 24.20	Beta	Ladabaum et al. [27]
Symptomatic presentation of regional CRC	40.00	36.00 - 44.00	Beta	Ladabaum et al. [27]
Symptomatic presentation of distant CRC	85.00	76.50 - 93.50	Beta	Veettil et al. [26]
LRA to S/P polypectomy	58.00	55.00 - 62.00	Beta	Veettil et al. [26]
HRA to S/P polypectomy	92.00	88.00 - 95.00	Beta	Veettil et al. [26]
Sensitivity of colonoscopy on CRC	94.70	90.40 - 97.20	Beta	Saini et al. [37]
Compliance to surveillance colonoscopy	60.00	30.00 - 100.00	Beta	Schoen et al. [22]
Compliance to berberine	80.00	30.00 - 100.00	Beta	Sandler et al. [24]
Risk rate, %				
Perforation due to colonoscopy	0.04	0.02 - 0.05	Beta	Lin et al. [28]
Major bleeding due to colonoscopy	0.08	0.05 - 0.14	Beta	Lin et al. [28]
Mortality, %				
Local CRC	3.27	2.73 - 3.90	Beta	Noone et al. [29]
Regional CRC	8.22	7.52 - 8.98	Beta	Noone et al. [29]
Distant CRC	46.93	45.49 - 48.39	Beta	Noone et al. [29]
Perforation	5.82	5.238 - 6.40	Beta	Gatto et al. [30]
Major bleeding events	6.00	1.00 - 16.00	Beta	Veettil et al. [31]
Cancer treatment	2.00	1.80 - 2.20	Beta	Ladabaum et al. [27]
Other cause	Age specific	NA	Beta	Parant A [32]
Costs (2022, US$)				
Berberine (300mg twice daily)	124.00	99.20 -148.80	Gamma	Local price
Commercial payments for persons under age 65				
Colonoscopy	1385.55	1108.44 - 1662.66	Gamma	Ladabaum et al. [34]
Colonoscopy with polypectomy	1853.59	1482.88 - 2224.31	Gamma	Ladabaum et al. [34]
Perforation due to colonoscopy	24834.22	19867.37 - 29801.06	Gamma	Ladabaum et al. [34]
Major bleeding due to colonoscopy	9164.61	7331.69 - 10997.53	Gamma	Ladabaum et al. [34]
CRC treatment by stage (age 50-65)				
Local, initial	43093.75	34475.00 - 51712.50	Gamma	Ladabaum et al. [34]
Local, continuing yearly	3428.45	2742.76 - 4114.14	Gamma	Ladabaum et al. [34]
Local, colorectal cancer death	77251.68	61801.35 - 92,702.02	Gamma	Ladabaum et al. [34]
Regional, initial	72510.85	58008.68 - 87,013.02	Gamma	Ladabaum et al. [34]
Regional, continuing yearly	4568.95	3655.16 - 5482.74	Gamma	Ladabaum et al. [34]
Regional, colorectal cancer death	81170.25	64936.20 - 97404.30	Gamma	Ladabaum et al. [34]
Distant, initial	94686.62	75749.30 - 113623.95	Gamma	Ladabaum et al. [34]
Distant, colorectal cancer death	108937.00	87149.60 - 130724.40	Gamma	Ladabaum et al. [34]
Costs, Medicare payments for persons age 65 and older				
Colonoscopy	786.27	629.01 - 943.52	Gamma	Ladabaum et al. [34]
Colonoscopy with polypectomy	1011.58	809.26 - 1213.90	Gamma	Ladabaum et al. [34]
Perforation due to colonoscopy	18396.58	14717.26 - 22,075.89	Gamma	Ladabaum et al. [34]
Major bleeding due to colonoscopy	6788.38	5430.71 - 8146.06	Gamma	Ladabaum et al. [34]
CRC treatment by stage (age 65 and older)				
Local, initial	31921.08	25536.86 - 38305.29	Gamma	Ladabaum et al. [34]
Local, continuing yearly	2539.98	2031.99 - 3047.98	Gamma	Ladabaum et al. [34]
Local, colorectal cancer death	57223.34	45778.67 - 68668.01	Gamma	Ladabaum et al. [34]
Regional, initial	53712.43	42969.94 - 64454.91	Gamma	Ladabaum et al. [34]
Regional, continuing yearly	3384.32	2707.46 - 64454.91	Gamma	Ladabaum et al. [34]
Regional, colorectal cancer death	60125.68	48100.54 - 72150.81	Gamma	Ladabaum et al. [34]
Distant, initial	70138.11	56110.49 - 84165.73	Gamma	Ladabaum et al. [34]
Distant, colorectal cancer death	80694.07	64555.26 - 96832.89	Gamma	Ladabaum et al. [34]
Health state utilities				
Non-CRC states	0.84	0.80 - 0.88	Beta	Ness et al. [35]
Local CRC	0.74	0.69 - 0.78	Beta	Ness et al. [35]
Regional CRC	0.67	0.62 - 0.72	Beta	Ness et al. [35]
Distant CRC	0.25	0.20 - 0.31	Beta	Ness et al. [35]
Colonoscopy(disutility)	0.0025	0.0023 - 0.0028	Beta	Saini et al. [36]
Annual discount rate, %	3.00	Fixed in PSA	NA	Haacker et al. [33]

Clinical and economic outcomes

The study measured the outcomes by lifetime costs in US dollars, quality-adjusted life-years (QALYs), and incremental cost-effectiveness ratio (ICER).

Sensitivity analyses

The study conducted one-way sensitivity analyses to estimate the effects of altering parameters. Costs were assumed to be varied by 20% and utilities and probabilities by 10% if 95% CI ranges were not available. A probabilistic sensitivity analysis (PSA) was also conducted to simultaneously examine the effects of all parameter uncertainties using a 100,000 Monte Carlo simulation via TreeAge Pro 2019. Furthermore, the study performed scenario analyses to assess model sensitivity to shorter or longer colonoscopy surveillance intervals after adenoma identification [38,39].

## Results

Base case analyses

Table 2 presents the results of modeling four strategies to prevent colorectal cancer in postpolypectomy patients. Treating patients with berberine alone was associated with mean costs of 37,480 and mean QALYs of 15.32 per patient. It was found to have greater incremental QALYs and lower incremental costs compared to no intervention, making it a dominant strategy compared to no intervention. The colonoscopy strategy produced mean costs of $16,391 and mean QALYs of 16.03 per patient, which dominates the berberine strategy. The addition of berberine to colonoscopy was associated with mean costs of $15,609 and mean QALYs of 16.05 per patient, resulting in a cost-saving and more additional QALYs gained compared to colonoscopy alone.

**Table 2:** Base case cost-effectiveness results of all the included strategies for preventing colorectal cancer given to the general population, aged 50 to 100 QALY - quality-adjusted life-year; ICER - incremental cost-effectiveness ratio

Strategy	No intervention	Berberine	Colonoscopy	Berberine + colonoscopy
Total costs ($US)	39,802	37,480	16,391	15,609
Total QALYs	14.66	15.32	16.03	16.05
Incremental cost($US)		Saving $2,322	Saving $21,089	Aaving $782
Incremental QALY		0.66	0.71	0.02
ICER compared to no intervention		Dominates	Dominates	Dominates
ICER compared to berberine			Dominates	Dominates
ICER compared to colonoscopy				Dominates

Sensitivity analyses

**Figure 2:**
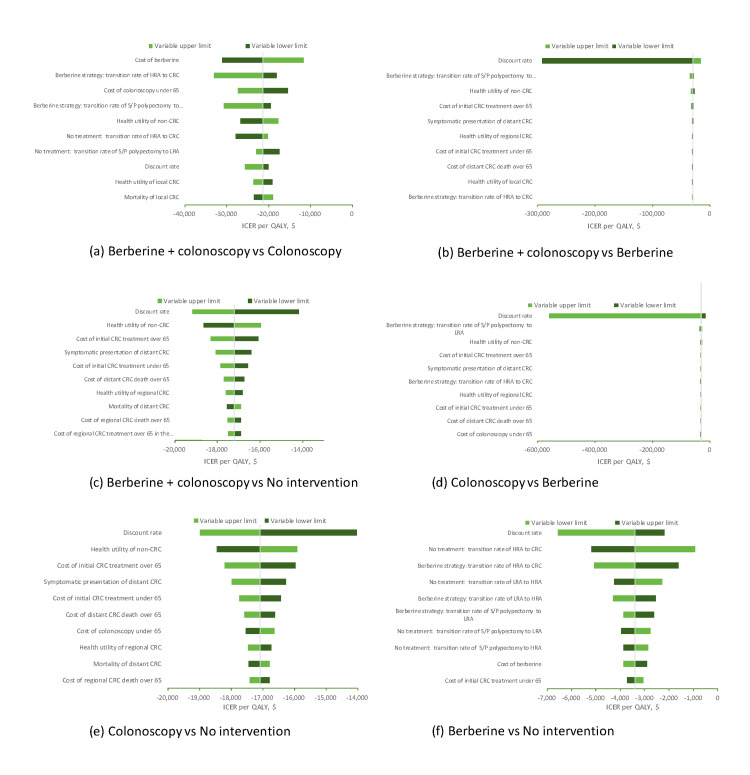
Tornado analysis of key variables for four strategies in postpolypectomy patients HRA - high-risk adenomas; LRA - low-risk adenomas; CRC - colorectal cancer; S/P - status post; QALY - quality-adjusted life-year; ICER - incremental cost-effectiveness ratio

Figure 2 shows key results from the one-way sensitivity analyses. The ICER between the addition of berberine to colonoscopy and colonoscopy alone was most sensitive to the cost of berberine, the transition probability of CRC from high-risk adenoma in the berberine group, and the cost of colonoscopy under 65. The combined strategy dominated colonoscopy alone in each of the tested variable limits. Berberine alone was not cost-effective at any tested variable lower or upper limits compared with either the addition of berberine to the colonoscopy regimen or colonoscopy alone. No intervention strategy was dominated in any case.

**Figure 3:**
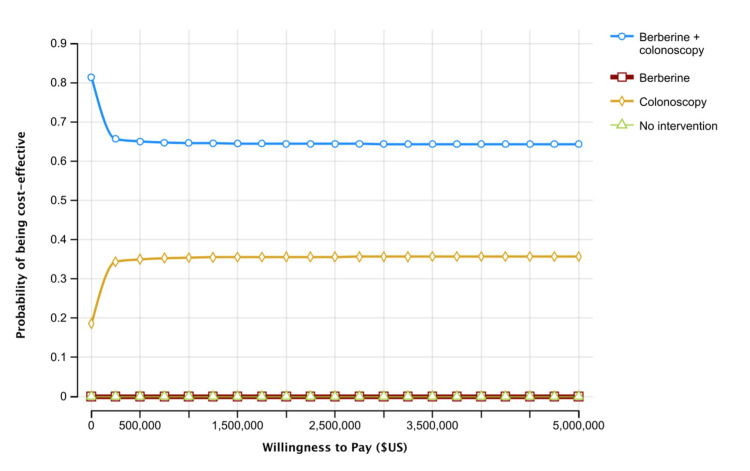
Acceptability curve of four strategies

The results of the PSA showed that the combined strategy and colonoscopy had around a 65 % and 35% chance of being cost-effective alternatives, respectively, when considering the generally acceptable WTP threshold of $100,000 per QALY (Figure 3). On the other hand, neither berberine nor intervention had any likelihood of being cost-effective compared to colonoscopy or the combined strategy.

In scenario analyses (Appendix), if patients with high-risk adenoma have a shorter colonoscopy surveillance interval at one year, the colonoscopy and the combined strategies were associated with lower costs and more QALYs gained than an interval at three years. In contrast, for those with lower-risk CRA or no CRA recurrence, the results suggested longer colonoscopy surveillance intervals than the base case, along with more cost-saving. Of note, the results were robust to the combined strategy's dominance or effectiveness compared to colonoscopy.

## Discussion

Due to a higher risk of recurrent CRA, post-polypectomy patients are twice as likely to be afflicted with CRC than the general population [40-42]. Emerging evidence has indicated that berberine effectively reduces the recurrence rate of CRA within postpolypectomy patients [16]. However, the economic value of berberine in the secondary prevention of CRC has not been evaluated. This study aimed to fill this gap and received significant results: The addition of berberine to surveillance colonoscopy is a cost-effective option compared to surveillance colonoscopy alone. Berberine alone was dominated by the combined strategy and colonoscopy alone, but it was cost-saving with more additional QALYs than no intervention.

Current evidence indicates that, compared to no intervention, berberine is a cost-saving strategy with additional benefits for preventing CRC in postpolypectomy patients. However, the berberine-alone strategy is unlikely to replace routine colonoscopy. Based on this model, adding berberine to surveillance colonoscopy is a cost-effective option, which is consistent with previous research on the effectiveness and cost-effectiveness of other chemoprevention agents. For instance, many clinical and cohort studies suggested that aspirin and calcium were likely to be cost-effective options for secondary prevention by effectively reducing CRA incidence [11, 43, 44]. However, the evidence is weak. With the increasing concerns of comparisons between prevention strategies, the cost-effectiveness analyses further found chemoprevention supplemented with colonoscopy resulted in more CRCs prevented and lives saved at a small increased cost [14, 45]. Besides, previous results indicated that, although chemoprevention effectively prevented CRC within both the general population and patients with a higher risk of CRC, it appeared less economically attractive for the general population [11, 44, 46]. Alternatively, it became more attractive to those higher-risk patients. As for berberine, the evidence of the primary prevention of CRC is limited. Further research is required to assess its long-term benefits and harms for the general population and the subgroup with a high risk of CRC.

The present study may underestimate the efficacy of berberine in preventing CRA, which is attributable to a marked disparity between the available evidence from the clinical trial and the data requirements for the microsimulation model. Specifically, the clinical trial is unable to provide the transition rates of low-risk to high-risk adenoma and high-risk adenoma to CRC due to a short follow-up period of two years. Those transition rates were assumed to be natural in both the berberine and placebo groups in the study, indicating a conservative assumption of the effectiveness of berberine in preventing CRC. For example, the transition rate from high-risk adenoma to CRC ranks second to influence the cost-effectiveness in one-way sensitivity analyses. Its potential underestimation may underrate the value of berberine. In addition, the trial did not point out at which time berberine took effect, and its protective effects stopped. Evidence is also limited to suggest extrapolation of treatment effects regarding another chemopreventive agent [47]. As with assumptions implied to explore the impact of aspirin, the present study assumed berberine is effective during drug exposure, and its protective effect does not persist after treatment cessation in the model [48]. This assumption may result in lower treatment effects due to ignoring potentially accumulated protective effects or additional benefits of berberine following treatment cessation [49].

Those findings were based on surveillance intervals suggested by current guidelines for colonoscopy surveillance [50]. When formulating the combination strategy, it is essential to consider whether surveillance colonoscopy intervals could be changed based on berberine when formulating the combination strategy. The present study further simulated scenarios to explore the effects of a less or more intensive surveillance colonoscopy combined with or without berberine after intervals in line with current guidelines (three to five year intervals based on polyp recurrence). When colonoscopy intervals were shortened to one year for the recurrence of high-risk adenoma, both the combination strategy and surveillance colonoscopy alone exhibited more excellent performance in cost-saving and effectiveness than routine intervals. Meanwhile, extending surveillance colonoscopy for the recurrence of low-risk CRA was also associated with cost-effective advantages regardless of the combination strategy and surveillance colonoscopy alone. In general, a combination strategy with a more intensive surveillance colonoscopy was superior to surveillance colonoscopy alone. For countries with limited health care resources, extending surveillance intervals to 10 years for patients with low-risk CRA recurrence may significantly reduce health costs at the expense of a slight QALY loss.

In addition, the present study also considered differential costs for patients aged <65 (reflecting commercial insurance payments) vs. those aged ≥65 (reflecting Medicare payments) when evaluating the cost-effectiveness of berberine chemoprevention. In the US, people usually change insurers over their lifetime, referring to private insurance when they are younger than 65 and Medicare when they are 65 and over [34]. Therefore, the present study simulated the costs based on Medicare payments, which is believed to reflect actual costs better than other measures in prior cost-effectiveness analyses regarding CRC chemopreventive agents.

In retrospect, the present study has several limitations. Firstly, the recurrence rate of low-risk to high-risk adenoma and high-risk adenoma to CRC was unavailable since no trials were conducted to evaluate berberine’s long-term clinical effectiveness in CRA recurrence and CRC reduction currently, increasing difficulty in assessing the efficacy of berberine. Secondly, the trial NCT02226185 provided primary clinical data for the study. As this trial mainly enrolled the Chinese population, bias may have existed due to the different characteristics of the Chinese and American patients. Thirdly, the present study did not compare the economic value of berberine to other chemoprevention agents. Fourthly, this model did not include the harms associated with berberine since no serious adverse events were reported. Further validation could focus on the long-term benefits and harms of berberine in terms of CRA and CRC recurrence, the observation from the US population, the outcome compared to another chemopreventive agent, and the constipation that may be caused when used in the elderly.

## Conclusions

Berberine shows promise as an agent for optimizing CRC prevention, as adding it to colonoscopy is the most cost-saving and the best buy option for secondary prevention of CRC among all four strategies. While berberine chemoprevention alone is dominated by surveillance colonoscopy, it is reasonable to consider using berberine chemoprevention for postpolypectomy patients in areas with limited healthcare access because it dominates no intervention strategy. Further research is needed to explore its long-term benefits and harms for patients with increased risk for CRC and the general population.

## Appendices

**Table 3:** Scenario analyses HRA - high-risk adenomas; LRA - low-risk adenomas.

Scenario for colonoscopy intervals	Strategy	Total costs ($)	Total QALYs	ICER
Scenario 2 3-year interval for LRA; 7-year interval for HRA; 7-year interval for no recurrence;	Colonoscopy	16,124	16.02	__
	Berberine + colonoscopy	15,357	16.04	Dominates
Scenario 3 3-year interval for LRA; 3-year interval for HRA; 3-year interval for no recurrence;	Colonoscopy	16,178	16.06	__
	Berberine + colonoscopy	16,545	16.09	12,235
Scenario 4 1-year interval for LRA; 3-year interval for HRA; 5-year interval for no recurrence;	Colonoscopy	15,756	16.36	419,873
	Berberine + colonoscopy	11,557	16.35	__
Scenario 5 1-year interval for LRA; 3-year interval for HRA; 3-year interval for no recurrence;	Colonoscopy	15,756	16.36	298,100
	Berberine + colonoscopy	12,775	16.35	__
Scenario 1 1-year interval for LRA; 5-yeas interval for HRA; 5-years interval for no recurrence;	Colonoscopy	14,982	16.34	__
	Berberine + colonoscopy	11,182	16.34	Dominates
Scenario 6 1-year interval for LRA; 5-year interval for HRA; 10-year interval for no recurrence;	Colonoscopy	14,982	16.34	__
	Berberine + colonoscopy	10,874	16.34	Dominates
Scenario 7 1-year interval for LRA; 10-year interval for HRA; 10-year interval for no recurrence;	Colonoscopy	14,116	16.34	176,600
	Berberine + colonoscopy	10,584	16.32	__
Scenario 8 3-year interval for LRA; 10-year interval for HRA; 10-year interval for no recurrence;	Colonoscopy	16,095	16.00	__
	Berberine + colonoscopy	15,426	16.02	Dominates
